# Clinical improvement of moderate depression with a combination of rhodiola and saffron in a non-student population: a double-blind, placebo-controlled study

**DOI:** 10.1192/j.eurpsy.2025.469

**Published:** 2025-08-26

**Authors:** P. Lemoine, C. Gal, E. Jacouton, S. Ait Abdellah

**Affiliations:** 1 Lyon University, Lyon; 2 Pileje Laboratoire, Paris, France

## Abstract

**Introduction:**

A number of medicinal plants have produced noticeable results in depression treatment including, *Rhodiola rosea* and *Crocus Sativus* (saffron).

**Objectives:**

To evaluate the efficacy and safety of a combination of rhodiola and saffron in patients with moderate depression.

**Methods:**

Adults with moderate depression (International Classification of Diseases [ICD-10]) of moderate intensity (Hamilton Depression Score [HAM-D] ≥ 17 and ≤ 23) were recruited (n=126; 64 students, 62 non students). Patients took 2 tablets of the supplement (i.e. 308 mg rhodiola and 30 mg saffron) or a placebo daily for 6 weeks. Main criterion was the evolution of HAM-D score between Day (D) 0 and D42. Other criteria were evolution of HAM-D, Hospital Anxiety and Depression Scale (HADS)-anxiety and HADS-depression, Patient’s Global Impression of Change (PGIC), Clinical Global Impression of severity and improvement (CGI-S and -I) at D0, D21 and D42, tolerability and compliance.

**Results:**

A significant 10-point decrease in HAM-D score was observed only in the supplemented non-student population between D0 and D21 (from 18.9±1.7 to 8.6±3.4 in Supplement group *versus* 18.5±1.8 to 11.2±4.2 in Placebo group, p=0.005). At D42, a 12-point decrease was observed in the Supplement group; the difference between the two groups being marginally significant (from 18.9±1.7 to 7.1±5.0 in Supplement group versus from 18.5±1.8 to 8.8±4.2 in Placebo group, p=0.087). The percentage of patients with a HAM-D score reduction >75% was higher in the Supplement group (p<0.05). The HAM-D score decrease was particularly marked in patients with HADS-anxiety ≥11 at D21. The distribution of patients by severity HAM-D classes (no, mild, moderate depression) was significantly different between the two groups (p<0.05 at D21 and D42), with a greater number of patients with no or mild symptoms in the Supplement group. The remission rate (HAM-D ≤ 7) was higher in this group (p<0.05 at D21 and D42). These results by classes were also found with HADS-depression scale. Results for CGI-S and CGI-I were in favor of the Supplement group (p<0.05 at D21). These results were not observed in students. Compliance and tolerability were good.

**Conclusions:**

As expected, the rhodiola plus saffron combination improved psychological well-being of non-student patients with moderate depression. The poor results observed in students suggest that health professionals should ascertain the real depressive state of students confronted with stressful and/or anxiety-provoking situations linked to exams, professional future or precarity. When prescribing, clinicians should not only rely on quantitative scales, but above all on their clinical feelings.

**Disclosure of Interest:**

None Declared

